# Membrane Systems for Biomedical Engineering

**DOI:** 10.3390/membranes13010041

**Published:** 2022-12-29

**Authors:** Ludomira H. Granicka, Wojciech Piątkiewicz

**Affiliations:** 1 Nalecz Institute of Biocybernetics and Biomedical Engineering, Polish Academy of Sciences, ul. Ks. Trojdena 4, 02-109 Warsaw, Poland; 2 PolymemTech sp. zo.o., ul. Wołodyjowskiego 46, 02-724 Warsaw, Poland

The thematic scope concerning membrane systems for biomedical engineering is very wide; it concerns new methods of designing membrane systems for biomedical and biomedical-related environmental processes. The aims of biomedical applications are metabolic support, the maintaining of tissue functions and the replacement of lost organs. Biomedical applications include the use of membrane nanosystems in process regulation, involving, among others, drug delivery systems and membrane scaffolds to support cell growth. 

Pathogenic contamination is considered to be a cause of waterborne diseases. Therefore, new materials have been explored as potential separation membranes for desalination and water purification with simultaneous antibacterial features. Moreover, the increasing number of antibiotic-resistant microorganism strains has resulted in the development of many approaches to obtaining effective antimicrobial materials. The membranes combining both functions, i.e., antibacterial activity and cell growth support, can be used, among others, in systems for dressing purposes. 

Membranes themselves as part of the systems intended for biomedical applications and environmental processes are the basis of this Special Issue, which is entitled “Membrane Systems for Biomedical Engineering”. This Special Issues focuses on methods of membrane formation; materials used for their production, e.g., natural materials; and composite materials, including nanoparticles, that are related to a large branch of nanotechnology. In addition, the practical aspects of membrane applications are discussed. This Special Issue opens with articles on the study of the systems of natural membranes for therapeutic purposes. 

In the study by de Oliveira et al. [1], a natural polyisoprene-based membrane was applied to induce the differentiation of adipose-tissue-derived mesenchymal stem cells to neuronal cells. Adipose-tissue-derived mesenchymal stem cells (ADMSCs) can differentiate into several cell lines, which makes them a promising option for regenerative medicine. For neuronal differentiation assessment, the cells seeded on polystyrene flasks coated with the applied membrane were characterized with flow cytometry, using immunocytochemical reactions, and with RT-PCR. After induction to neuronal differentiation, the ADMSCs formed neurospheres composed of neural precursors. The authors demonstrated that the applied membrane induced differentiation of ADMSCs to neural precursors without the addition of neurogenic growth factors or gene transfection, suggesting that the cells’ differentiation mechanisms are related to their mechanotransduction capacity regulated by YAP and AMOT proteins. The applied technique using the polyisoprene-based membrane can be applied to the treatment of neurodegenerative diseases. 

A natural polyisoprene-based membrane was also applied to induce the differentiation of human mesenchymal stem cells (hMSCs) from the human umbilical cord to cholinergic-like neurons. Stricker et al. [2] demonstrated the possibility of obtaining neurospheres without applying growth factors or gene transfection via immobilization of undifferentiated hMSCs on a natural biopolymer membrane coating in substrate. The obtained results indicate the possibility of developing a safe method to repair the loss of cholinergic neurons. Moreover, it may be of use in preclinical models of Alzheimer’s disease before translation.

In the study conducted by Dziedzic et al. [3], a decellularized human amniotic membrane (DAM) for application in bone tissue engineering was evaluated. A new strategy of grafting the bone and periodontal lesion area with an amniotic membrane, adipose-derived stromal cells, and a mineralized extracellular matrix was investigated with the aim of stimulating bone deposition in a defect on a rodent model. Using microcomputed tomography and histological analysis, the authors observed that the decellularized amniotic membrane with adipose-derived stromal cells or without cells and the mineralized extracellular matrix ensured bone tissue healing. Moreover, the membrane supported neovascularization and promoted osteoconduction. The authors recommended the DAM as a potential scaffold carrier of cells and the extracellular matrix in tissue engineering applications. 

Antidrug resistance—the ability of bacteria, parasites, and viruses to resist antibiotics and antivirals—leads to the inability to prevent infections. To counter this problem, several attempts to develop new materials or modify existing ones by involving nanocomposites have been made. Metallic nanoparticles, due to their unique properties, can be applied as antimicrobials. 

Deokar et al. [4] described a comparative analysis of immobilized on cotton metallic nanoparticles (NPs) synthetized in water and in ethanol. The antibacterial effect was assessed using Staphylococcus aureus and Escherichia coli strains. CuO NP-coated bandages in water or ethanol were found to be more effective against both *S. aureus* and *E. coli* than ZnO NPs-coated bandages. Moreover, it was demonstrated that ethanol can be replaced by water for the synthesis of metal-oxide NPs, which could be a great way to avoid ignition accidents during the bulk-scale synthesis of metal-oxide NPs in commercial companies. In addition, in vivo experiments indicated that water-based metal-oxide-coated bandages were less toxic than ethanol-based ones. As elaborated by the authors, coated bandages could be an efficient tool in combating hospital-acquired infections. 

Some attention has been paid to wound dressing, which plays a vital role in post-operative aftercare, and it is necessary to develop dressings for applications on the border of soft and hard tissues. Grzeczkowicz et al. [5] studied multifunctional polyelectrolyte-layered membrane nanocomposites enhanced, among others, by hydroxyapatite nanoparticles and gold nanoparticles, and/or fullerenol to achieve a wound dressing that could be applied on the bone–skin interface. The obtained results indicate that the designed bilayer membranes prevent the internalization of bacteriostatic elements caused by human osteoblastic cells, simultaneously ensuring the proper morphology of the cells maintained within. Therefore, they can be recommended for usage as multilayer membranes elements of dressings at the bone–skin interface.

The review paper by Kwiatkowska et al. [6] presents subjectively selected nanomaterials used in wound dressings, including metallic nanoparticles (NPs), and it refers to the aspects of their application as antimicrobial factors. This literature review is supplemented with the results of the team’s research on the elements of multifunctional new-generation dressings containing nanoparticles.

Another approach to wound healing is the application of derivatives of amniotic membranes. Schmiedova et al. [7] reviewed the therapeutic potential of amniotic membrane grafts, which have been applied for the treatment of chronic wounds, burns, and dermal injuries.

Mahar et al. [8] summarized in their review the recent progress of nitride (MXene)-based membranes for water purification and antibacterial applications in theoretical and experimental aspects. Different fabrication and modification methods for MXene laminates were highlighted. 

Zieliński et al. [9] presented the hybrid approach (i.e., numerical–physical approach) toward the assessment of membrane usability for biomedical purposes. Computer models, however useful for biomedical education and research, are unfit for medical device testing. The chosen approach to the modeling of the cardiovascular system and/or respiratory system was discussed by the authors with particular emphasis given to the membrane-based hybrid cardiopulmonary simulator (the artificial patient), which was elaborated by the authors.

The membrane systems for biomedical engineering applications constitute a very wide area of research. In this Special Issue, we compile important contributions to membrane research in the aspects of tissue regeneration, organ function preservation, and antimicrobial function, presenting the possibility of filling the gap between research and commercialization.

In conclusion, the editors would like to thank the authors and reviewers for their valuable contributions to this Special Issue and the editorial staff of “Membrane Systems for Biomedical Engineering” for their valuable support.

## Data Availability

Not applicable.
